# An Incidental Finding of Gastric Mucosa-Associated Lymphoma (MALToma) in a Sleeve Gastrectomy with Literature Review

**DOI:** 10.1155/2020/8855127

**Published:** 2020-12-03

**Authors:** Brenda Mai, Michaelangelo Friscia, Pamela Younes, Jaiyeola Thomas-Ogunniyi, Lei Chen

**Affiliations:** Department of Pathology and Laboratory Medicine, McGovern Medical School at the University of Texas Health Science Center, 6431 Fannin Street, Houston, TX, USA

## Abstract

Mucosal-associated lymphoid tissue (MALT) lymphoma can typically be identified as a mass lesion or nodularity endoscopically and macroscopically. We report an incidental finding of a *Helicobacter pylori* gastric mucosal-associated lymphoid tissue (MALT) lymphoma in a sleeve gastrectomy specimen with no gross examination findings.

## 1. Introduction

The macroscopic examination of specimens and the selection of representative sections for subsequent microscopic examination are crucial and fundamental to the accurate diagnoses in pathology. William Grossman claims that in over 90% of specimens, an accurate diagnosis can be made with just the gross examination only [1]. The decline of autopsies and the rise in reliance on pathologists' assistants (PAs) for handling and processing specimens have resulted in a decrease in exposure to gross findings for pathologists and residents in training [2]. However, the gross examination and description can greatly aid in diagnoses and reveal new incidental lesions that are important to patient outcomes. We report an incidental finding of a gastric mucosal-associated lymphoid tissue (MALT) lymphoma in a sleeve gastrectomy specimen.

## 2. Case Presentation

**Figure 1:**
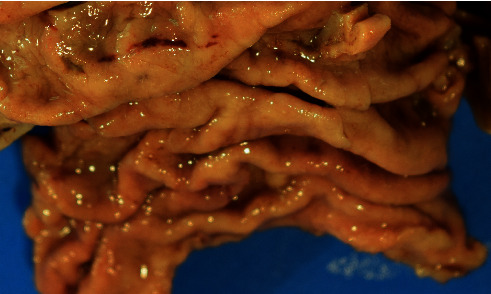
Sleeve gastrectomy specimen with focal area of petechial hemorrhage.

**Figure 2:**
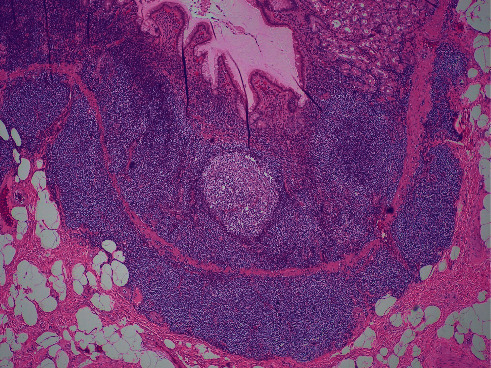
Atypical lymphoid aggregates expanding the lamina propria (hematoxylin and eosin; 40*x*).

**Figure 3:**
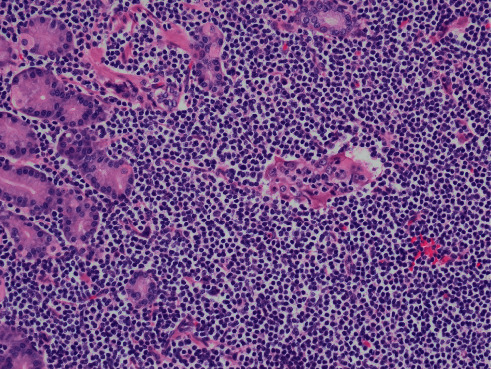
Glandular destruction secondary to the neoplastic lymphoid cells (hematoxylin and eosin; 200*x*).

This case report describes a 54-year-old male who underwent a laparoscopic sleeve resection procedure for morbid obesity. Prior to the procedure, the patient had a weight of 115.9 kilograms and a body mass index (BMI) of 49.77 and had failed both diet and medical weight management. His other medical history included hypertension, dyslipidemia, and sigmoid diverticulosis. His surgical history comprised of a laparoscopic cholecystectomy and an open reduction internal fixation of fracture on his ankle. He has a family history of diabetes mellitus type 2 in both parents. The patient admitted to drinking alcohol weekly but denied tobacco and recreational drug use. Physical examination did not reveal lymphadenopathy or hepatosplenomegaly. Preoperative evaluation of the peripheral blood was within normal limits with no evidence of a hematological malignancy or lymphoproliferative process. In addition, the patient did not have any positive findings on review of systems or any symptoms. Preoperative gastroduodenoscopy did not reveal any lesions; however, two antral biopsies were taken which revealed mild chronic inactive gastritis. A “sleeve gastrectomy specimen” was received in the pathology department. On gross examination, the gastric mucosa was described as tan-pink, smooth, and glistening with a focal area of hemorrhage (Figure 1). There were no masses, no nodularities, no ulceration, or abnormal rugal folds. Representative sections of the hemorrhagic region were taken, along with several routine random areas of normal-appearing mucosa. Other than the focal area of hemorrhage, there was little evidence to indicate an underlying pathological process or malignancy, as small hemorrhagic areas are common findings in surgical specimens. On histological examination, sections taken from the hemorrhagic areas showed a diffuse atypical lymphoid infiltrate extending into the lamina propria and submucosa, with nodular pattern and focal germinal centers (Figure 2). Proliferation of plasma cells was also evident. In addition, there was an associated architectural distortion with gastric glandular atrophy (Figure 3). Immunohistochemical stains with the appropriate controls were obtained revealing diffuse sheets of lymphoma cells that were positive for CD20, CD43, and BCL-2. They were negative for CD3, CD5, CD23, CD138, BCL-6, and Cyclin D1. The plasma cells were monoclonal with kappa restriction. The proliferation index by Ki-67 was low, approximately 1–5%, except for the reactive germinal centers. *Helicobacter (H.) pylori* stain on three of the blocks was negative. With these histological findings, the patient was diagnosed with a MALToma. Positron emission tomography (PET) scan was performed which revealed a lesion in the sigmoid colon. Subsequently, the patient underwent another round of biopsies which showed mild chronic inactive gastritis within the stomach and benign lymphoid aggregates in the colon. The patient was placed on omeprazole, clairthromycin, and amoxicillin. And, six-month follow-up established endoscopic and histologic remission.

## 3. Discussion

This is not the first case report of an incidental MALT lymphoma in a sleeve gastrectomy, as several other investigators have identified a MALT lymphoma in other bariatric specimens [3–6]. A literature review found 4 other case reports with a MALToma diagnosed within a sleeve gastrectomy specimen (Table 1). There was no sex predilection as there were 3 males and 2 females. The average age at diagnosis was 47 years of age with an age range of 28 to 59. The BMIs averaged 49.1 with a range of 45 to 60. One of the cases did not comment on the gross examination of the sleeve gastrectomy specimen, whereas the remaining 3 cases reported a mass ranging in size from 1.2 to 18 cm. The *H. pylori* status was available for all but 1 of the cases and they were negative.

Interestingly, this case report was the only case report that had no significant gross findings, whereas all the other cases reported identified a mass lesion in the specimen. A recent article by AbdullGaffar et al. challenges the need for routine microscopic evaluation of bariatric sleeve gastrectomy specimens [7]. Their prospective study identified 546 specimens and reported that there was no malignancy and less than five of the specimens had benign incidental findings. The researchers emphasize a thorough macroscopic evaluation only of these specimens to save time and cost, as incidental malignant findings are rare. A metanalysis study by Zullo et al. combined the data from 38 manuscripts with a total of over 2000 patients with MALTomas and concluded that MALTomas had the following endoscopic findings: 52.1% ulcerative pattern, 23.5% hypertrophic rugal folds, 12.7% normal or hyperemic mucosa, 9.7% exophytic mass, and 1% petechial hemorrhage. We would like to comment that although incidental findings are rare, they are still significant and greatly impact patient care and outcomes [8]. In addition, with the rising epidemic in obesity and bariatric procedures, we suggest that the practice of gross examination, proper sampling techniques, and microscopic examination be preserved for bariatric specimens.

In conclusion, our case emphasizes the importance of examining surgical specimens both macroscopically and microscopically for the identification of incidental malignant findings in asymptomatic patients. Although a large majority of MALTomas can be identified with gross lesions endoscopically and macroscopically on the specimen, a small percent of MALTomas have no gross findings. It is imperative to perform representative sampling on these specimens.

## Figures and Tables

**Table 1:** Incidental MALToma in sleeve gastrectomy samples.

	This case	De Silva et al.	Kopach et al.	Quesada et al.	Helman et al.
Age	54	59	39	53	28
Sex	M	F	M	M	F
BMI	49.77	45	44.8	60	46
Gross findings	Focal hemorrhage	18 × 3 cm mass	No information	8 cm mass	1.8 × 1.2 cm mass
*H. pylori*	Negative	Negative	Negative	No information	Negative
